# Armenian and Syrian Relief Fund

**Published:** 1919-01

**Authors:** 


### Armenian and Syrian Relief Fund.

On account of the influenza epidemic and weather conditions the $30,000,000 campaign for Armenian and Syrian Relief funds has been postponed to February 3-10 for the states of the Southern Military Department—Texas, Oklahoma, Arkansas, Louisiana, New Mexico and Arizona.

Several late cablegrams received by Southwestern District Headquarters from relief workers and Red Cross Agencies in western Asia and Europe make urgent calls for food, clothing and agricultural supplies in the war devastated areas of the Turkish Empire. One month's delay in sending relief means 20,000 deaths, says Williams S. Nelson, American Consular Agent at Tripoli, Syria; and not more than one-quarter of the existing Armenian population will survive until next harvest with present relief resources.

The American Committee for Relief in the Near East estimates that a generous oversubscription of the $30,000,000 is needed to rescue the starving peoples of Armenia, Syria, Palestine, Northwestern Persia, and the Russian Caucasus, and to tide them over to the period of self support. With the signing of the armistice and the probable freedom of these subject races from the Turkish yoke, relief work can now go on unhindered.

---

Sixty dollars will save a life. How many will you save? Armenian Relief Campaign, February 3-10.

